# Loss-of-function of *MEDIATOR 12* or *13* subunits causes the swelling of root hairs in response to sucrose and abscisic acid in Arabidopsis

**DOI:** 10.1080/15592324.2023.2191460

**Published:** 2023-03-21

**Authors:** Javier Raya-González, José Carlos Prado-Rodríguez, León Francisco Ruiz-Herrera, José López-Bucio

**Affiliations:** a Facultad de Químico Farmacobiología, Universidad Michoacana de San Nicolás de Hidalgo, Morelia, Michoacán, México; b Instituto de Investigaciones Químico Biológicas, Universidad Michoacana de San Nicolás de Hidalgo, Morelia, México

**Keywords:** *Arabidopsis thaliana*, root hair development, abscisic acid, sucrose, mediator complex

## Abstract

Root hairs are epidermal cell extensions that increase the root surface for water and nutrient acquisition. Thus, both the initiation and elongation of root hairs are critical for soil exploration and plant adaptation to ever changing growth conditions. Here, we describe the critical roles of two subunits of the Mediator complex, MED12 and MED13, in root hair growth in response to sucrose and abscisic acid, which are tightly linked to abiotic stress resistance. When compared to the WT, *med12* and *med13* mutants showed increased sensitivity to sucrose and ABA treatments on root meristem and elongation zones that were accompanied with alterations in root hair length and morphology, leading to the isodiametric growth of these structures. The swollen root hair phenotype appeared to be specific, since *med8* or *med16* mutants did not develop rounded hairs when supplied with 4.8% sucrose. Under standard growth medium, MED12 and MED13 were mainly expressed in root vascular tissues and cotyledons, and their expression was repressed by sucrose or ABA. Interestingly, *med12* and *med13* mutants manifested exacerbated levels of nitric oxide under normal growth conditions, and upon sucrose supplementation in trichoblast cells, which coincided with root hair deformation. Our results indicate that MED12 and MED13 play non-redundant functions for maintenance of root hair integrity in response to sucrose and ABA and involve nitric oxide as a cellular messenger in *Arabidopsis thaliana*.

## Introduction

In multicellular organisms, cell fate determination is controlled by environmental conditions and cell-specific cues that concertedly generate the distinct tissues.^1^ In *Arabidopsis* roots, files of epidermal cells differentiate into root hairs that act as extensions influencing both soil physicochemical conditions through exudation of a wide variety of compounds and enhance absorptive potential.^2^

Root hair length and density are key factors for plant growth and development owing its direct involvement in water and nutrient acquisition.^3–5^ In the *Arabidopsis* root, there are two kinds of epidermal cell types, root-hair cells (also named H cells or trichoblasts), and non-hair cells (N cells or atrichoblasts), which are arranged depending upon external and internal factors, including nutrients such as phosphorus and nitrogen, water availability, phytohormones, and signaling through reactive nitrogen species (RNS) or reactive oxygen species (ROS), which operate through regulation of transcription factors.^3–7^

The identification of *Arabidopsis* mutants defective on root hair initiation or elongation led to the cloning of the GLABRA (GL2) transcription factor that is expressed in atrichoblasts and blocks ROOT HAIR DEFECTIVE (RHD6) activity to generate the N cell identity.^8^ RHD6 and its homologue RHD6-LIKE (RSL), two basic helix-loop-helix (bHLH) transcription factors, have been described as key regulators for root hair outgrowth.^9^ Repression of GL2 allows RHD6 to activate downstream transcriptional components to start root hair formation.^9^ The phytohormones auxin and ethylene and several microorganisms known to produce auxins or auxin-like compounds trigger root hair growth.^5,10,11^ Consistently, reduced elongation of root hairs occurs in *Arabidopsis* mutants affected in auxin signaling or transport, including *slr1/iaa14*, *aux1*, *arf7arf19*, *axr3*, and *tir1*.^8,12–14^ The lack of root hairs in *rhd6* mutants can be restored by auxin or ethylene, suggesting that these phytohormones act downstream of RHD6.^15^

Abscisic acid (ABA) is a major phytohormone orchestrating the plant resistance to abiotic stress, including water deficit, salinity and drought.^16^ ABA application reduces root hair length and activates the differentiation of trichoblasts, increasing root hair density, which was accompanied with an induction of nitric oxide (NO) within the hair protrusion.^6^ This suggests that ABA and NO act as key modulators to regulate root hair development. A molecular mechanism by which ABA controls root hair formation came from the identification of DNA BINDING WITH ONE FINGER (DOF)-type transcriptional regulator, OBF BINDING PROTEIN (OBP4), whose overexpression inhibits cell elongation and control root hair development in *Arabidopsis*.^17^ ABA application triggers the accumulation of OBP4 protein, which negatively regulates the expression of ROOT HAIR DEFECTIVE-LIKE (RSL2), a helix-loop-helix transcription factor involved in root hair elongation.^17^

MEDIATOR (MED) is a multi-protein, transcriptional complex ubiquitous to eukaryotes including plants. In *Arabidopsis*, it is composed by 30 subunits, and organized in four modules; the core, the head, the tail, and a dissociable cyclin kinase module, called CDK8.^18^ MED subunits are involved in multiple processes, including embryo and leaf development, fertility, root morphogenesis, mineral nutrition, and pathogen resistance.^19–25^ MED12 and MED13 belong to the CDK8 module playing non-redundant roles in embryo patterning, vegetative and floral transitions, and flowering regulation.^19,20,25^ Loss-of-function of either MED12 or MED13 produced a short root phenotype, with more lateral roots in response to auxin and sugar supplementation.^21^ However, whether MED12 and MED13 may influence epidermal cell differentiation, and their relationship with plant hormones or second messengers remains unknown.

In this report, we analyzed the phenotypes of roots and root hairs in a suite of MED mutants including *med8*, *med12*, *med13*, and *med16* exposed to either ABA or sucrose. Detailed cellular and structural analyses revealed that sucrose or ABA application to *med12* and *med13* mutants, reduced cell elongation and caused root hair swelling, which appeared to be specific, since *med8* or *med16* mutants did not manifest these alterations in response to sucrose. Mutant root hairs entered isodiametric growth which correlated with high nitric oxide levels as detected by confocal microscopy. Sucrose and ABA repressed *MED12* and *MED13* expression in both the shoot and root systems in a dose-dependent manner, positioning these proteins in the signal transduction cascade for epidermal cell elongation mediated by sucrose and ABA in *Arabidopsis*.

## Materials and methods

### Plant material and growth conditions

*Arabidopsis thaliana* WT (Col-0), the transgenic *Arabidopsis* lines *pMED12:GUS* and *pMED13:GUS* were used to assess *MED12* and *MED13* expression, respectively, according to Gillmor et al. (2010).^19^ The mutant lines, *cct-2/crp-3/med12* ^19,25^, *cct-3/crp-4/med13* ^19,25^, *med8* (SALK_092406)^22^, and *med16–2* (SALK_048091)^24^ were employed for comparisons with the WT phenotype. To grow *in vitro*, seeds were disinfected with 95% (v/v) ethanol for 5 min and 20% (v/v) bleach for 7 min. After careful washing in distilled water, the seeds were stratified for 48 h at 4°C, and germinated and grown on agar plates containing 0.2× Murashige and Skoog (MS) basal salt mixture (PhytoTech Labs), lacking vitamins, and supplemented with 17.52 mM sucrose, referred as control condition. Plates were placed vertically at an angle of 65° to allow root growth along the agar surface and to enable proper shoot growth. Plates were placed in a plant growth chamber (Percival AR-95 L) with a photoperiod of 16 h of light/8 h darkness, light intensity of 300 μmol/m^−2^/s^−1^ and temperature of 22°C.

### Propidium iodide staining

Root tips and root hair morphology were analyzed via propidium iodide (PI) staining, which marks viable cells and tissue files in red color. Twelve-day-old *Arabidopsis* seedlings were incubated in 10 mg ml^−1^ of PI solution for 1 min, carefully washed and mounted in 1:1 glycerol/water proportion on microscope slides. The sample was recorded at wavelengths specific to PI fluorescence with a 568 nm excitation line and emission window of 585–610 nm, using a confocal microscope (Olympus FV1000 equipped with an objective lens Olympus PlanFlour N40× and a digital camera).

### Detection of nitric oxide

The detection of nitric oxide (NO) in the primary root tip and root hairs was performed using the specific probe 4,5-diaminofluorescein diacetate (DAF-2 DA), which freely diffuses through root cells and is useful for NO detection. The seedlings were incubated in 10 µM DAF-2 DA for 30 min in darkness, washed 3 times with sterilized, distilled water and placed on slides. Fluorescence was detected using an Olympus FV1000 confocal microscope, and quantified with the Image J program and expressed in arbitrary units (AU).

### Histochemical analysis

Detection of β-glucuronidase (GUS) activity in plant tissues was performed by incubating overnight *Arabidopsis* seedlings at 37°C in a GUS reaction solution, which contains the enzymatic substrate (0.5 mg ml^−1^ 5-bromo-4-chloro-3-indolyl-β-D-glucuronide) and 100 mM sodium phosphate, pH 7.0). Seedlings were cleared through 60 min incubation into 0.2 M HCl/20% methanol at 63°C, subsequently transferred into 1.75 M NaOH/60% ethanol solution by 30 min at room temperature, and finally, dehydrated by 40%, 20%, and 10% (v/v) ethanol dilutions for 20 min each at room temperature^26^. Seedlings were mounted with glycerol 50% on microscope slides and visualized via the Nomarsky optics in a LEICA DM500B microscope. The vasculature and root tips, as well as the leaves of at least 10 stained seedlings expressing *pMED12:GUS* and *pMED13:GUS*, were recorded and analyzed.

### Data analysis

The data were statistically analyzed using the STATISTICA 12.0 program (Dell, StatSoft, Austin, Texas, USA). Significant differences among different traits and treatments of WT (Col-0) seedlings and mutants were determined by univariate and multivariate analyzes with Tukey’s post hoc tests. Different letters were used to indicate means that differed significantly (*p* < 0.05) and were placed over the corresponding standard error bars.

## Results

### MED12 and MED13 regulate cell elongation and differentiation in response to sucrose and ABA

**Figure 1.**
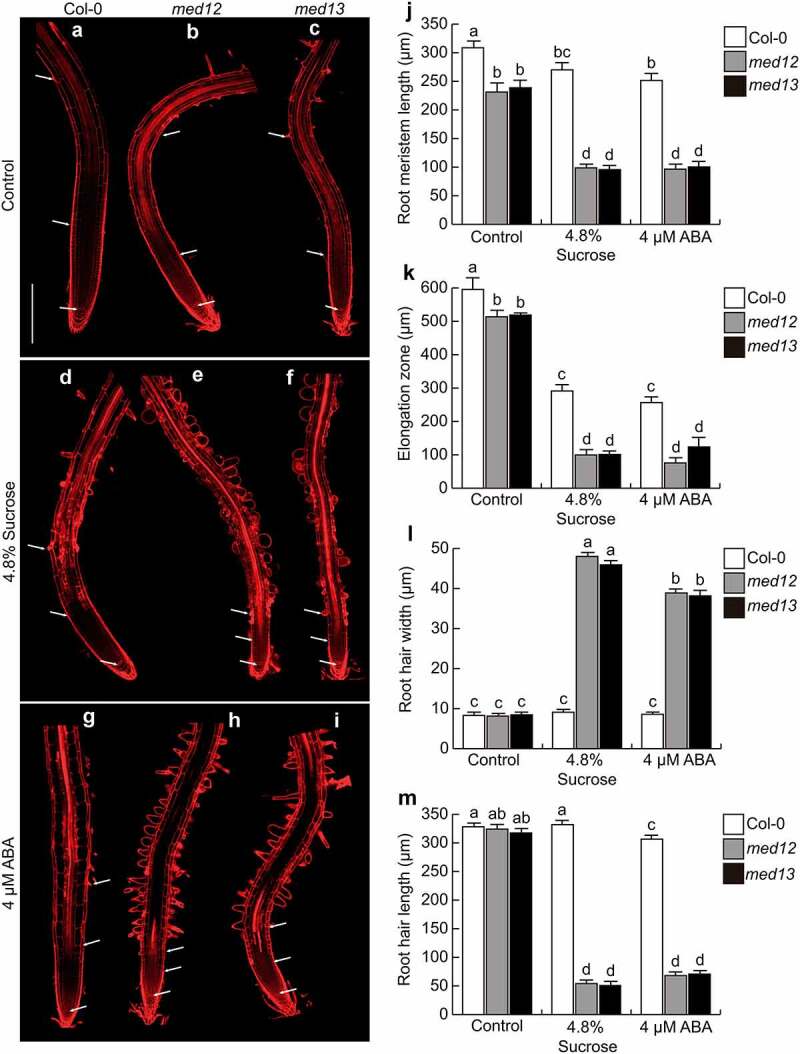
*MED12* or *MED13* loss-of-function affects root meristem size and root hair development. Wild type (Col-0), *med12* and *med13 Arabidopsis* seedlings were germinated and grown in 0.2× Murashige and Skoog (MS) media or media supplemented with either 4.8% Sucrose or 4 µM ABA for 12 days. (a-i) Representative micrographs of at least ten WT, *med12* and *med13* root stained with propidium iodide and visualized by confocal microscopy. (j) Root meristem length. (k) Elongation zone. (l) Root hair width. (m) Root hair length. White arrows indicate elongation and meristematic zones. Scale bar in a = 200 µm. Error bars with different letters represent statistical differences (*P*= ˂0.05). The experiment was repeated three times with comparable results.

ABA and sugars interact to control different plant developmental processes and abiotic stress-related responses.^27^ To investigate whether MED12 and MED13 could play a role in cell elongation and differentiation mediated by ABA and sucrose within the *Arabidopsis* primary root, we applied 4.8% sucrose or 4 µM ABA to the growth medium of wild-type (WT; Columbia-0; Col-0), and *med12* or *med13* single mutants. As previously reported by Raya-González et al. (2017)^21^, under standard growth conditions, *med12* and *med13* mutants had shorter primary roots, primary root meristem, and cell elongation zone than WT plants (Figure 1a-c,j,k). Interestingly, these effects were accompanied with changes in root hair development. In WT seedlings, the sucrose or ABA treatments applied did not affect root hair morphology or structure, since they were comparable to control conditions (Figure 1l-m), but drastically influenced both root hair length and width in *med12* and *med13* mutants (Figure 1a-i,l-m). These results suggest the critical role for MED12 and MED13 to configure root hair morphogenesis in *Arabidopsis thaliana*.

### Mutation of MED12 and MED13 led to the isodiametric growth of root hairs in response to sucrose and ABA

**Figure 2.**
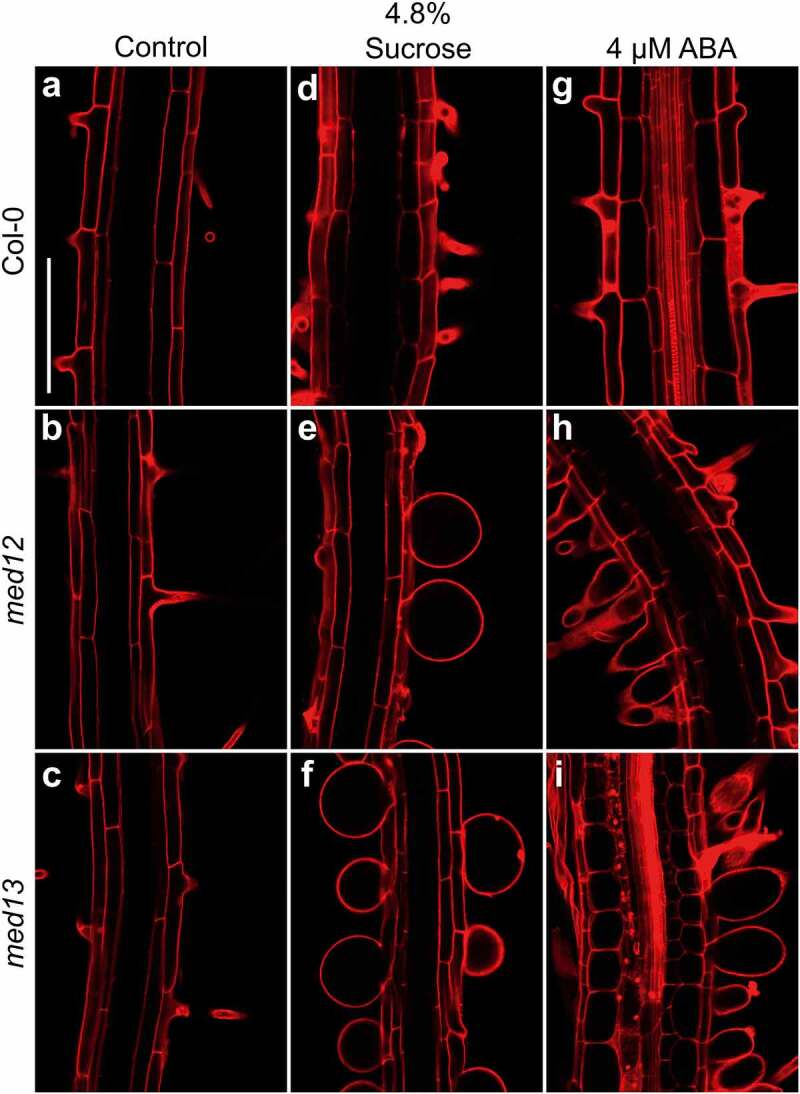
Effects of sucrose and ABA on root hair morphology in WT, *med12* and *med13* seedlings. Wild type WT (Col-0), *med12* and *med13* seedlings were germinated and grown for 12 d on 0.2× MS media or media supplemented with 4.8% sucrose or 4 µM ABA. Roots were stained with propidium iodide and visualized by confocal microscopy. (a-i) Representative micrographs of root hairs in WT, *med12*, and *med13* seedlings at the indicated treatments. Scale bar in a = 100 µm. This experiment was repeated three times with similar results.

**Figure 3.**
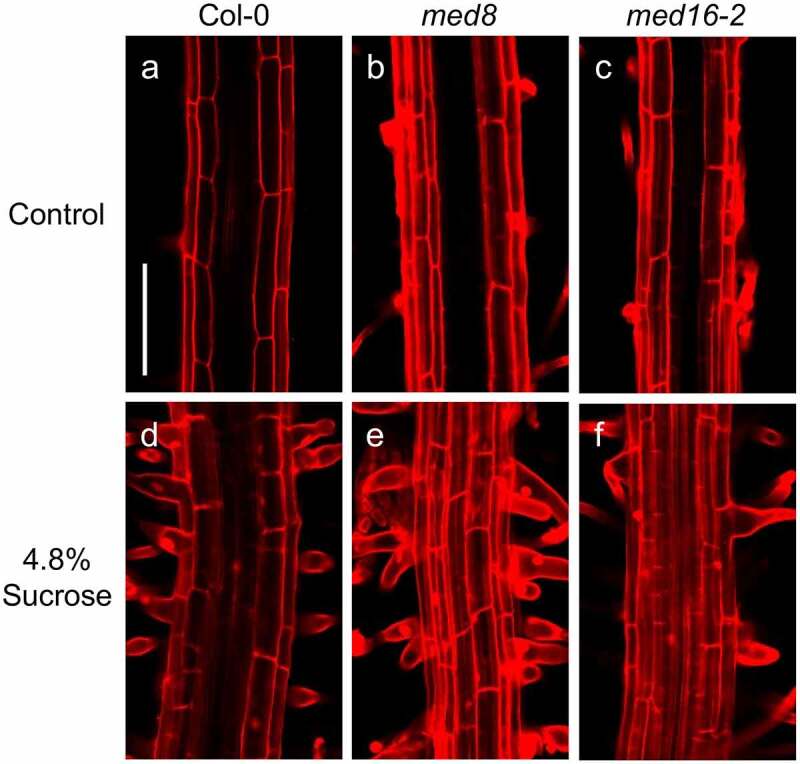
Effects of sucrose on root hair morphology in WT, *med8* and *med16* seedlings. Wild type WT (Col-0), *med8* and *med16* seedlings were germinated and grown for 12 d on 0.2× MS media or media supplemented with 4.8% sucrose. Roots were stained with propidium iodide and visualized by confocal microscopy. (a-f) Representative micrographs of root hairs in WT, *med8*, and *med16* seedlings at the indicated treatments. Each panel shows representative photographs of at least 10 seedlings analyzed. Scale bar in a = 100 µm. This experiment was repeated two times with similar results.

The swollen root hairs in *med12* and *med13* mutants prompted us to study the viability of these cells in the corresponding lines stained with propidium iodide (PI), which freely penetrates into the cytoplasm of cells upon damage.^23^ Confocal microscopy analysis of the root hair forming zone showed that ABA or sucrose induced the nearly isodiametric growth of root hairs in *med12* and *med13* mutants without affecting cell viability (Figure 2a-i). The formation of rounded root hairs of *med12* and *med13* mutants in response to sucrose treatments appeared to be specific, since mutation of *MED8* or *MED16* did not trigger this bulbous cell phenotype (Figure 3a-f). These data show not only the specificity of the root hair phenotype of *med12* and *med13* mutants but also the integrity of these cells under the applied treatments.

### MED12 and MED13 modulate NO accumulation in Arabidopsis roots

**Figure 4.**
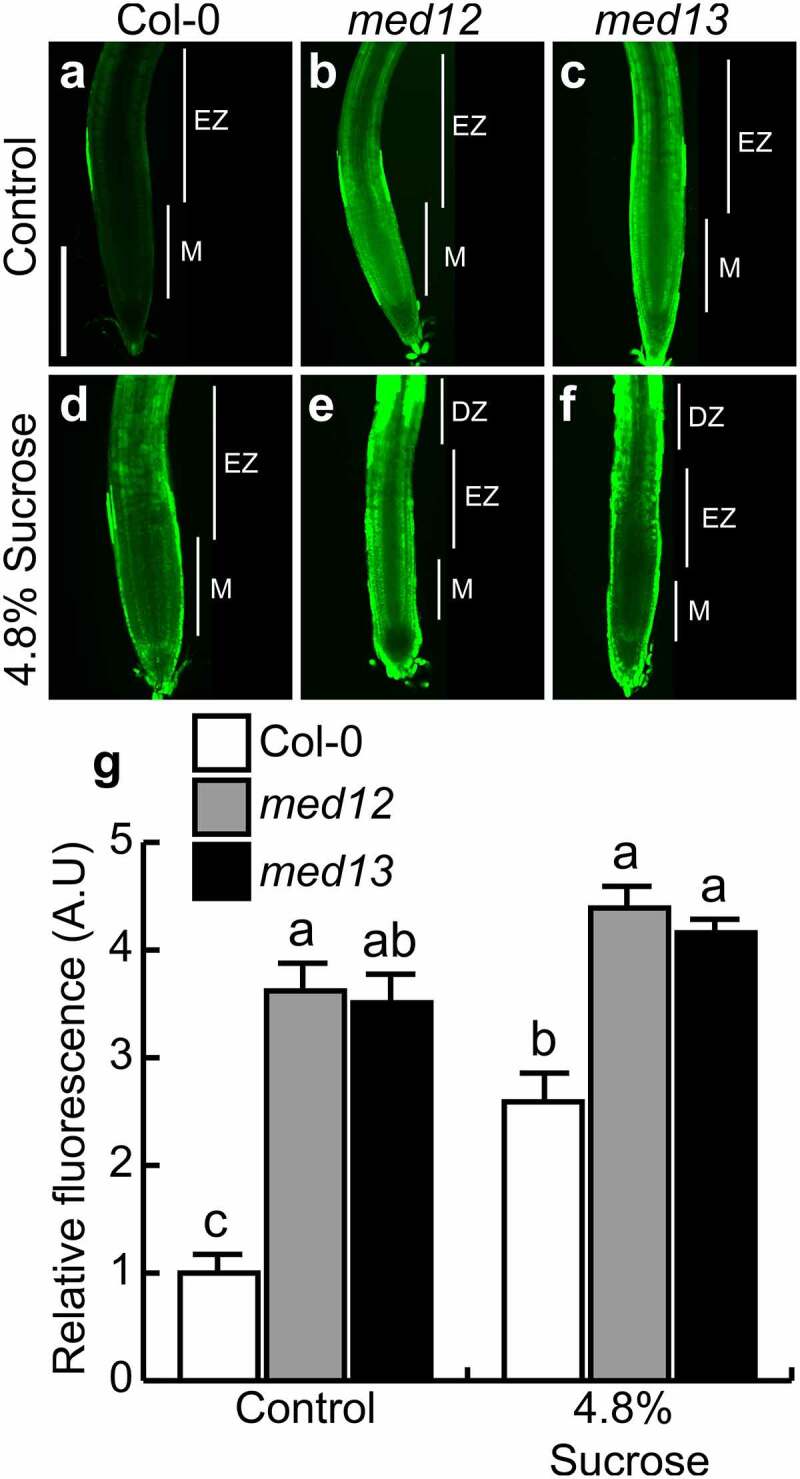
MED12 and MED13 influence nitric oxide production at the root tip. Detection of nitric oxide in the root tips of WT (Col-0), *med12* and *med13* seedlings germinated and grown for 12 d on 0.2× MS media or media supplemented with 4.8% sucrose . (a-f) Representative images of nitric oxide accumulation by using the specific probe DAF-2 DA. (g) Representative plot of nitric oxide accumulation in root tips expressed as relative fluorescence. M: Meristem, EZ: Elongation zone, DZ: Differentiation zone. Scale bar = 100 µm. The standard error with different letters indicates statistical differences (*P*= ˂ 0.05.). The experiment was repeated three times with comparable results.

Nitric oxide (NO) is a second messenger involved in distinct plant development processes. The accumulation of NO in primary root tips, lateral roots, adventitious roots, and root hairs is a hallmark of organogenesis.^6,28,29^ To determine whether NO signaling could be regulated by MED12 and MED13 and analyze their relationship with sucrose responses, NO was visualized in primary roots by using the fluorescent probe 4, 5-diaminofluorescein diacetate (DAF-2 DA), which freely penetrates through the cell membrane and is hydrolyzed by esterases in the cytoplasm to produce 4,5-diaminofluorescein (DAF-2). DAF-2 specifically reacts with NO to produce a triazole compound, triazolofluorescein (DAF-2T).^30^ Under our growth conditions, NO could be detected at the epidermis, close to the elongation zone in WT root tips, whereas *med12* and *med13* mutants showed up to four-fold higher NO accumulation, evidenced by a strong green fluorescence (Figure 4a-c,g). Interestingly, 4.8% sucrose triggers NO production and accumulation in primary root tips in WT plants, whereas in *med12* and *med13* mutants, NO accumulation was comparable with the control condition (Figure 4a-g). NO was mainly accumulated at the elongation and differentiation zones, where root hair formation takes place (Figure 4a-g). This suggests that sucrose induces NO accumulation and that MED12 and MED13 orchestrate this process.

### Sucrose induces NO accumulation in trichoblasts

**Figure 5.**
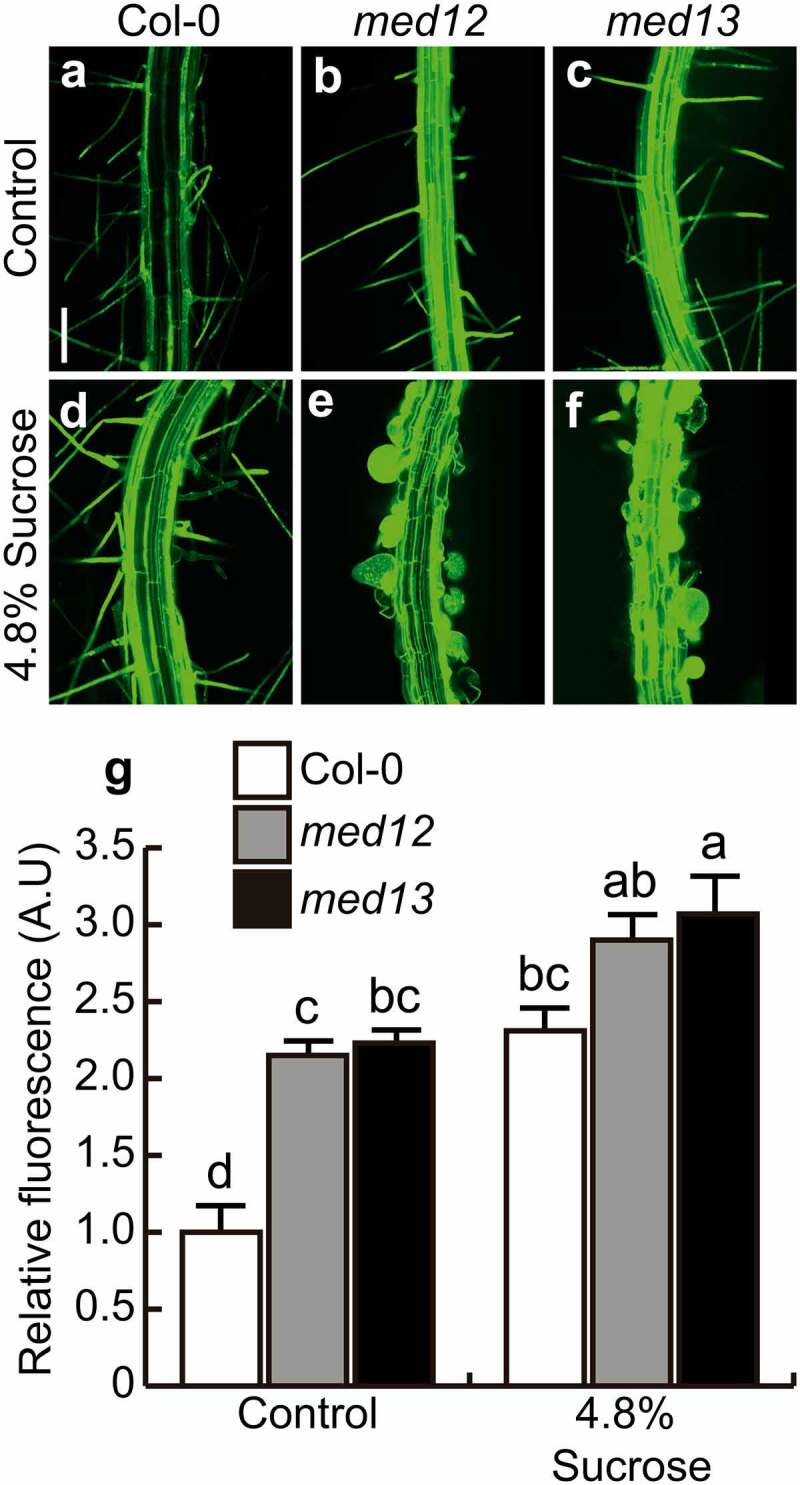
MED12 and MED13 negatively control nitric oxide production at the differentiation zone. (a-f) Confocal images of at least ten seedlings analyzed showing detection of nitric oxide through the specific probe DAF-2 DA in seedlings germinated and grown in Petri plates with 0.2× MS medium supplemented or not with 4.8% sucrose 12 d after germination. (g) Nitric oxide determinations represented as relative fluorescence from the whole root. Scale bar in a = 100 µm. The standard error with different letters indicates statistical differences (*P=* ˂0.05). This experiment was repeated three times with comparable results.

**Figure 6.**
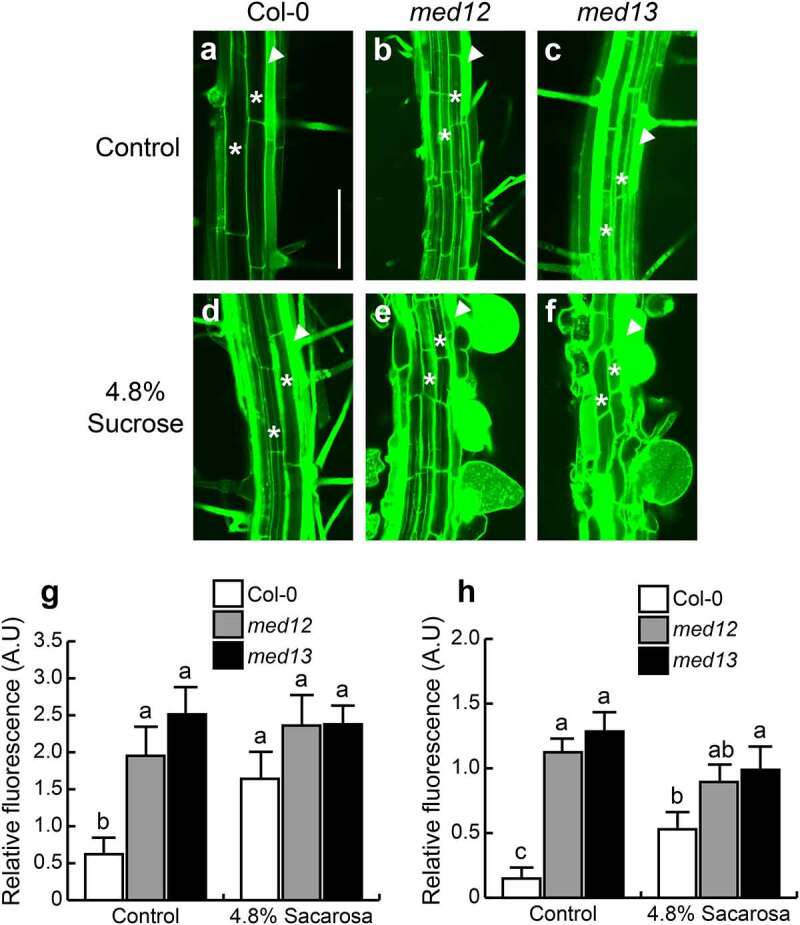
Sucrose triggers nitric oxide accumulation in trichoblasts and atrichoblasts in WT, *med12* and *med13* seedlings. (a-f) Confocal images showing NO accumulation in trichoblasts (arrow heads) and atrichoblasts (asterisks) in WT (a, d), *med12* (b, e) and *med13* (c, f) roots through the specific probe DAF-2 DA in seedlings germinated and grown 12 d in Petri plates with 0.2× MS medium in control conditions (a-c) or in response to 4.8% sucrose (d-f). (g, h) Nitric oxide determinations represented as relative fluorescence in trichoblasts (g) and atrichoblasts (h). Scale bar in a = 100 µm. The standard error with different letters indicates statistical differences (*P=* ˂0.05). This experiment was repeated three times with comparable results.

The results described above suggest that NO could mediate root hair development in *med12* and *med13* mutants in response to sucrose. We next evaluated NO accumulation in root hairs in 12 d-old WT, *med12* and *med13* seedlings. Under standard growth conditions, NO levels were higher in trichoblast cells than in atrichoblast cells (Figure 5a). In contrast *MED12* or *MED13* mutation leads to much more detection of NO in trichoblast cells, which could be phenocopied by sucrose application in the WT (Figure 5a-d). Indeed, the round root hairs developed in *med12* and *med13* in response to sucrose had the strongest green fluorescence, indicating highest NO levels (Figures 5a-g, 6). These data show the correlation between NO and the root hair phenotype upon mutation of the Mediator complex subunits 12 and 13 in *Arabidopsis*.

### Sucrose and ABA repress MED12 and MED13 expression in Arabidopsis

**Figure 7.**
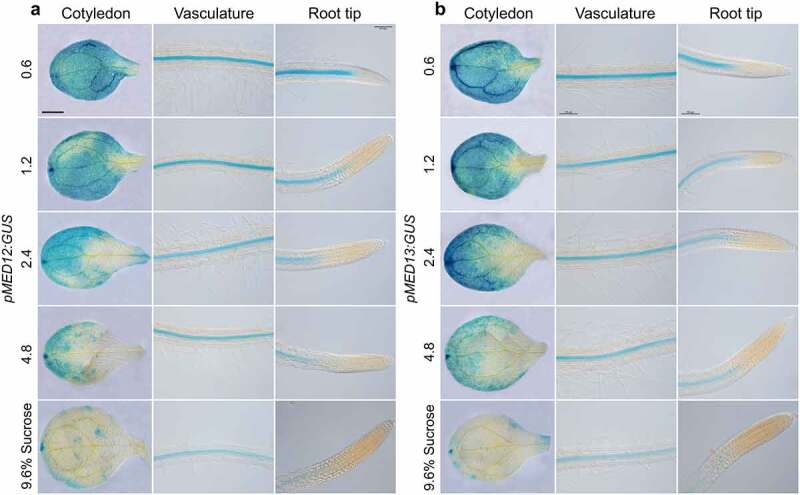
Effect of sucrose on *MED12* and *MED13* expression. Wild-type (WT) transgenic seedlings harboring the *pMED12:GUS* and *pMED13:GUS* gene constructs were germinated and grown in petri plates with 0.2× MS medium supplemented or not with 0.6, 1.2, 2.4, 4.8, and 9.6% sucrose. (a-b) Representative micrographs of *MED12* and *MED13* expression in cotyledon, vasculature and root tip for the indicated treatments. Scale bar in a = 100 µm. The experiment was repeated twice with comparable results.

**Figure 8.**
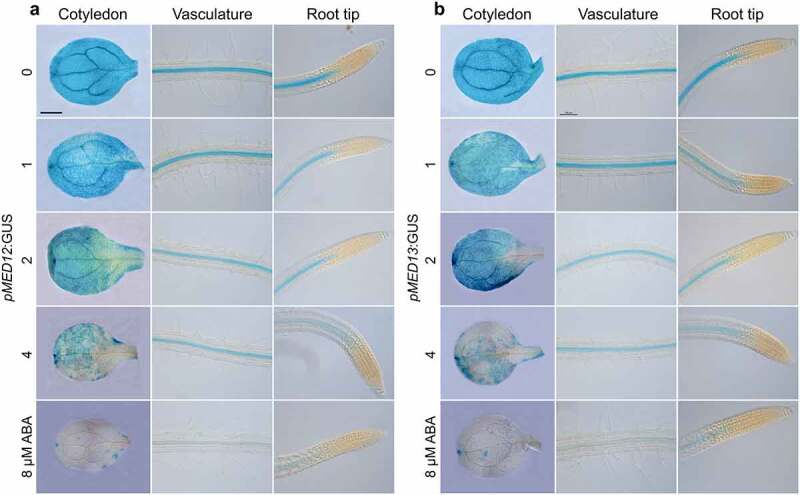
ABA represses *MED12* and *MED13* expression in *Arabidopsis*. WT (Col) wild-type transgenic seedlings harboring the *pMED12:GUS* and *pMED13:GUS* gene constructs were germinated and grown in petri plates with 0.2× MS medium supplied with 0, 1, 2, 4, and 8 µM ABA. (a-b) Representative micrographs of MED12 and MED13 expression in cotyledon, vasculature and root tip at the indicated treatments. Scale bar in a = 100 µm. The experiment was repeated twice with comparable results.

*MED12* and *MED13* could be involved in sucrose and ABA responses in epidermal cell differentiation. To determine this possible interaction, we analyzed the effect of sucrose on *MED12* and *MED13* expression, by using *Arabidopsis* transgenic plants, which express the *pMED12:GUS* and *pMED13:GUS* gene constructs.^31^ For this purpose, *pMED12:GUS* and *pMED13:GUS Arabidopsis* seedlings were germinated and grown for 7d in MS 0.2× media supplemented with 0.6%, 1.2%, 2.4%, 4.8% and 9.6% sucrose and their expression patterns analyzed in three different regions of seedlings, including cotyledons, the root differentiation zone, and the primary root tip. Under 0.6% sucrose, *MED12* and *MED13* expression was preferentially located in vascular cells of cotyledons and roots (Figure 7a, b). Interestingly, sucrose clearly repressed MED12 and MED13 expression in a dose-dependent manner (Figure 7a, b). Next, we tested the effects of 0, 1, 2, 4 and 8 µM ABA on the expression of *pMED12:GUS* and *pMED13:GUS* transgenes. As the concentration of ABA increased, *MED12* and *MED13* expression decreased in the cotyledon and vasculature of seedlings (Figure 8a, b), indicating its repressing role on transcriptional expression of both MED12 and MED13 subunits. Together, these results suggest that sucrose and ABA signaling negatively regulates *MED12* and *MED13* expression in *Arabidopsis* seedlings.

## Discussion

The Mediator complex is a multi-subunit co-activator of transcription in eukaryots, for which MED12 and MED13, two CDK subunits have been implicated in distinct plant development processes. Previously, we reported the functions of MED12 and MED13 in primary root growth of *Arabidopsis thaliana* seedlings and the growth response to sugars.^21^ In this study, we went further to analyze the relationship of MED12 and MED13 with ABA and sucrose responses on root hair growth, an aspect of great importance for plants regarding the roles of these epidermal cells in nutrient and water uptake.

Root hairs are extensions of epidermal cells termed trichoblasts and the genetics of root hair initiation and elongation has been investigated for decades, leading to the identification of many genes implicated in these processes in the model plant *Arabidopsis thaliana*. Here, we found that *Arabidopsis* WT seedlings germinated and grown under high sucrose (4.8%) or ABA (4 µM) supplements were weakly affected. However, the primary roots of *med12* and *med13* mutants showed increased sensitivity to these compounds, because the root meristem and cell elongation zone of the primary root were drastically reduced, implying MED12 and MED13 in ABA and sucrose responses for primary root elongation. However, upon a microscopical inspection of the mature zone of primary roots of WT, *med12* and *med13* seedlings, we observed the nearly isodiametric growth of the root hairs that was absent in other Mediator mutants such as *med8* and *med16*. These data suggest that the formation of swollen root hairs is a *MED12* and *MED13* loss-of-function specific response to both sucrose and ABA.

Sucrose not only acts as a carbon source but also as a signaling input alone or in coordination with plant hormones, including ABA, to regulate gene expression.^32,33^ In this context, we found that application of ABA to the plant growth media led trichoblasts to have much wider diameters that disturbs cell length and changes both the size and shape of the root hairs. Together, these data suggest that sucrose and ABA pathways could be interacting to control root hair development via a MED12-MED13-dependent mechanism in *Arabidopsis*. Root hair growth requires fine regulation at molecular level and involves polar cell wall-loosening activity, which drives the elongation of the hair bulge. Interestingly, reduction of cellulosic and non-cellulosic polysaccharide synthesis leads to the isodiametric growth of trichoblasts.^34–37^ Loss-of-function in KOJAK (*KJK*), *SEVERAL RADIAL SWELLING* (*SRS*), and *PROCUSTE1* (*PRC1*) genes phenocopy the *med12* and *med13* phenotype in plants exposed to sucrose or ABA.^34–36^ This suggests that MED12 and MED13 could be involved in cell wall organization perhaps acting directly on the expression of the above mentioned genes, and this possibility merits further investigation.

Root hair tip growth is a highly dynamic process requiring well-organized cytoskeleton through the coordination of actin filaments and microtubules to move organelles and vesicles.^38,39^ Previous reports described that ROP members of the Rho family of GTPases are involved in the configuration of actin filaments and microtubules in leaf cell morphogenesis and in root tip growth.^40–42^ Transgenic expression of the constitutively active CA1 form of ROP2 caused alterations on root hair growth and development through disruption of cell polarity.^40–42^ Interestingly, in CA1 seedlings grown with 5% sucrose, about 30% of root hairs were bulbous, whereas at 1% sucrose no bulbous root hairs were evident.^43^ A comparable phenotype in root hairs was found in *med12* and *med13* mutants in response to 4.8% sucrose. This suggests that MED12 and MED13 could act in actin filament and microtubule organization, which enables polar growth at the root hair tip.

Second messengers, such as nitric oxide (NO), play key roles in epidermal cell differentiation.^44^ During root-hair-growth process, NO is produced and accumulated in high levels in trichoblasts, which is required for endocytosis, vesicle formation and trafficking.^7,45^ We found that sucrose triggers a strong NO accumulation in WT, *med12*, and *med13* roots, mainly in root hair cells, and these effects were exacerbated in the mutants. This shows a new role for MED12 and MED13 in coordinating NO levels in response to sucrose and ABA on root epidermal cells. Indeed, sucrose or ABA treatments drastically reduced cell size and root apical meristem length in *med12* and *med13* mutants, which was accompanied with defects in root hair structure and morphology and higher NO detection within these growth zones, but particularly in bulbous root hairs, in which NO strongly accumulated. A recent report by Lombardo and Lamattina (2018) demonstrated that ABA could trigger an increase of NO in root hairs^7^. Both ABA and NO deviated the orientation of microtubules from their longitudinal axis in control roots, to an oblique orientation upon ABA or NO treatments. In principle, these previous findings suggest that *MED12*/*MED13* mutation and consequently, NO accumulation may affect the formation of root hairs through modifying cytoskeletal dynamics.

Previous studies on the regulation of MED gene expression showed that a large number of their subunits modulate transcription in response to ABA, which may be explained by the presence of two functional *cis*-acting elements, ACGT and SOSEM within their promoters^46^. However, whether ABA modulates *MED12* and *MED13* transcription remained unknown. Our analysis of gene expression revealed that sucrose and ABA negatively regulate *MED12* and *MED1*3 expression in *Arabidopsis* cotyledons and in the root vasculature. Being MEDIATOR of fundamental importance for transcription, it was of interest to find that either *MED12* or *MED13* are preferentially expressed in the cotyledons and root vasculature. This raised the question why the root hairs located at the epidermis manifest aberrant phenotypes if no expression is observed at this cell layer. Considering the strengths and possible limitations of using *pMED12:GUS* and *pMED13:GUS* analyses, we find plausible that *pMED12* and *pMED13* activity in epidermis and cortex may be below the limit of detection at the times assayed.

The repression of both subunits in cotyledons and vasculature was comparable and occurred in a dose-dependent manner in response to sucrose or ABA, which may affect the overall arrangement and functionality of the MED complex. Recently, sucrose application to the growth media negatively regulated the sucrose transporter SUC2 in the primary root tip and leaves.^47^ SUC2 expression domain was found in sieve elements (SE) and phloem of the roots.^48^ As shown in the current report, *MED12* and *MED13* expression pattern is mainly located in vascular tissues of roots in a comparable manner to SUC2, opening the possibility that MED12 and MED13 could regulate sucrose-transport and/or response to control root system configuration. The fact that other MED subunits, including *med8* and *med16* did not manifest root hair swelling upon sucrose or ABA treatments, indicate that some subunits play specific functions in response to different stimuli.

## Data Availability

The datasets generated during the current study are available from corresponding authors upon reasonable request.

## References

[1] Yu Q, Li P, Liang N, Wang H, Xu M, Wu S. Cell-fate specification in Arabidopsis roots requires coordinative action of lineage instruction and positional reprogramming. Plant Physiol. 2017;175(2):816–12. doi:10.1104/pp.17.00814. 28821591 PMC5619903

[2] Gumil S, Dunand C. Cell growth and differentiation in Arabidopsis epidermal cells. J Ext Bot. 2007;58(14):3829–3840. doi:10.1093/jxb/erm253. 18162628

[3] Gilroy S, Jones D. Through form to function: root hair development and nutrient uptake. Trends Plant Sci. 2000;5(2):56–60. doi:10.1016/S1360-1385(99)01551-4. 10664614

[4] López-Bucio J, Cruz-Ramírez A, Herrera-Estrella L. The role of nutrient availability in regulating root architecture. Curr Opin Plant Biol. 2003;6(3):280–287. doi:10.1016/S1369-5266(03)00035-9. 12753979

[5] Ortiz-Castro R, Contreras-Cornejo HA, Macias-Rodríguez LM, López-Bucio J. The role of microbial signals in plant growth and development. Plant Signal Behav. 2009;4(8):701–712. doi:10.4161/psb.4.8.9047. 19820333 PMC2801380

[6] Raya-González J, López-Bucio JS, López-Bucio J . Nitric oxide and hydrogen peroxide in root organogenesis. Nitric oxide and hydrogen peroxide signaling in higher plants. 1st ed. Springer Cham; 2019; p. 157–173.

[7] Lombardo MC, Lamattina L. Abscisic acid and nitric oxide modulate cytoskeleton organization, root hair growth and ectopic hair formation in Arabidopsis. Nitric Oxide. 2018;80:89–97. doi:10.1016/j.niox.2018.09.002. 30236618

[8] Menand B, Yi K, Jouannic S, Hoffmann L, Ryan E, Linstead P, Schaefer DG, Dolan L. An ancient mechanism controls the development of cells with a rooting function in land plants. Science. 2007;316:1477–1480. doi:10.1126/science.1142618. 17556585

[9] Shibata M, Sugimoto K. A gene regulatory network for root hair development. J Plant Res. 2019;132(3):301–309. doi:10.1007/s10265-019-01100-2. 30903397 PMC7082380

[10] Contreras-Cornejo HA, Macias-Rodríguez L, Cortés-Penagos C, López-Bucio J. *Trichoderma virens*, a plant beneficial fungus, enhances biomass production and promotes lateral root growth through an auxin-dependent mechanism in Arabidopsis. Plant Physiol. 2009;149(3):1579–1592. doi:10.1104/pp.108.130369. 19176721 PMC2649400

[11] Paque S, Weijers D. Auxin: the plant molecule that influences almost anything. BMC Biol. 2016;14(1):67. doi:10.1186/s12915-016-0291-0. 27510039 PMC4980777

[12] Rahman A, Hosokawa S, Oono Y, Amakawa T, Goto N, Tsurumi S. Auxin and ethylene response interactions during Arabidopsis root hair development dissected by auxin influx modulators. Plant Physiol. 2002;130(4):1908–1917. doi:10.1104/pp.010546. 12481073 PMC166701

[13] Okushima Y, Overvoorde PJ, Arima K, Alonso JM, Chan A, Chang C, Ecker JR, Hughes B, Lui A, Nguyen D, et al. Functional genomic analysis of the AUXIN RESPONSE FACTOR gene family members in *Arabidopsis thaliana*: unique and overlapping functions of ARF7 and ARF19. Plant Cell. 2005;17(2):444–463. doi:10.1105/tpc.104.028316. 15659631 PMC548818

[14] Li SB, Xie ZZ, Hu CG, Zhang JZ. A review of auxin response factors (ARFs) in plants. Front Plant Sci. 2016;7:47. doi:10.3389/fpls.2016.00047. 26870066 PMC4737911

[15] Masucci JD, Schiefelbein JW. The *rhd6* mutation of *Arabidopsis thaliana* alters root-hair initiation through auxin-and-ethylene-associated process. Plant Physiol. 1994;106(4):1335–1346. doi:10.1104/pp.106.4.1335. 12232412 PMC159671

[16] Aslam MM, Wassem M, Jakada BH, Okal EJ, Lei Z, Saqib HAS, Xu W, Zhang Q, Zhang Q. Mechanisms of abscisic acid-mediated drought stress responses in plants. Int J Mol Sci. 2022;23(3):1084. doi:10.3390/ijms23031084. 35163008 PMC8835272

[17] Rymen B, Kawamura A, Schafer S, Breuer C, Iwase A, Shibata M, Ikeda M, Mitsuda N, Koncz C, Ohme-Takagi M, et al. ABA suppresses root hair growth via the OBP4 transcriptional regulator. Plant Physiol. 2017;173(3):1750–1762. doi:10.1104/pp.16.01945. 28167701 PMC5338652

[18] Kidd BN, Cahill DM, Manners JM, Schenk PM, Kazan K. Diverse roles of the Mediator complex in plants. Semin Cell Dev Biol. 2011;22(7):741–748. doi:10.1016/j.semcdb.2011.07.012. 21803167

[19] Gillmor CS, Park MY, Smith MR, Pepitone R, Kerstetter RA, Poethig RS. The MED12-MED13 module of Mediator regulates the timing of embryo patterning in Arabidopsis. Development. 2010;137(1):113–122. doi:10.1242/dev.043174. 20023166 PMC2796935

[20] Gillmor CS, Silva-Ortega CO, Willman MR, Buendía-Monreal M, Poethig RS. The Arabidopsis Mediator CDK8 module genes CCT (MED12) and GCT (MED13) are global regulators of developmental phase transitions. Development. 2014;141:4580–4589. doi:10.1242/dev.111229. 25377553 PMC4302938

[21] Raya-González J, López-Bucio JS, Prado-Rodríguez JC, Ruiz-Herrera LF, Guevara-García AA, López-Bucio J. The MEDIATOR genes MED12 and MED13 control Arabidopsis root system configuration influencing sugar and auxin responses. Plant Mol Biol. 2017;95(1–2):141–156. doi:10.1007/s11103-017-0647-z. 28780645

[22] Raya-González J, Ortiz-Castro R, Ruiz-Herrera LF, Kazan K, López-Bucio J. PHYTOCHROME and FLOWERING TIME1/MEDIATOR25 regulates lateral root formation via auxin signaling in Arabidopsis. Plant Physiol. 2014;165(2):880–894. doi:10.1104/pp.114.239806. 24784134 PMC4044844

[23] Raya-González J, Oropeza-Aburto A, López-Bucio JS, Guevara-García AA, De Veylder L, López-Bucio J, Herrera-Estrella L. MEDIATOR18 influences Arabidopsis root architecture, represses auxin signaling and is a critical factor for cell viability in root meristems. Plant J. 2018;96(5):909–985. doi:10.1111/tpj.14114. 30270572

[24] Raya-González J, Ojeda-Rivera YO, Mora-Macias J, Oropeza-Aburto A, Ruiz-Herrera LF, López-Bucio J, Herrera-Estrella L. MEDIATOR 16 orchestrates local and systemic responses to phosphate scarcity in Arabidopsis roots. New Phytol. 2021;229(3):1278–1288. doi:10.1111/nph.16989. 33034045

[25] Imura Y, Kobayashi Y, Yamamoto S, Furutani M, Tasaka M, Abe M, Araki T. CRYTIC PRECOCIOUS/MED12 is a novel flowering regulator with multiple target steps in Arabidopsis. Plant Cell Physiol. 2012;53(2):287–303. doi:10.1093/pcp/pcs002. 22247249 PMC3278046

[26] Malamy JE, Benfey PN. Organization and cell differentiation in lateral roots of *Arabidopsis thaliana*. Development. 1997;124:33–44. doi:10.1242/dev.124.1.33. 9006065

[27] Cheng WH, Endo A, Zhou L, Penney J, Chen H-C, Arroyo A, Leon P, Nambara E, Asami T, Seo M, et al. A unique short-chain dehydrogenase/reductase in *Arabidopsis* glucose signaling and abscisic acid biosynthesis and functions. Plant Cell. 2002;14(11):2723–2743. doi:10.1105/tpc.006494. 12417697 PMC152723

[28] Méndez-Bravo A, Raya-González J, Herrera-Estrella L, López-Bucio J. Nitric oxide is involved in alkamide-induced lateral root development in Arabidopsis. Plant Cell Physiol. 2010;51(10):1612–1626. doi:10.1093/pcp/pcq117. 20685967

[29] Barrera-Ortíz S, Garnica-Vergara A, Esparza-Reynoso S, García-Cardenas E, Raya-González J, Ruiz-Herrera LF, López-Bucio J. Jasmonic acid-ethylene crosstalk via ETHYLENE INSENSITIVE 2 reprograms Arabidopsis root system architecture through nitric oxide accumulation. J Plant Growth Regul. 2018;37(2):438–451. doi:10.1007/s00344-017-9741-3.

[30] Kojima H, Nakatsubo N, Kikuchi K, Kawahuara S, Kirino Y, Nagoshi H, Hirata Y, Nagano T. Detection and Imaging of Nitric Oxide with Novel Fluorescent Indicators: diaminofluoresceins. Anal Chem. 1998;70(13):2446–2453. doi:10.1021/ac9801723. 9666719

[31] Toro-De León G D, García-Aguilar M, Gillmor CS. Non-equivalent contributions of maternal and paternal genomes to early plant embryogenesis. Nature. 2014;514(7524):624–627. doi:10.1038/nature13620. 25209660

[32] Price J, Li TC, Kang SG, Na JK, Jang JC. Mechanisms of glucose signaling during germination of Arabidopsis. Plant Physiol. 2003;132(3):1424–1438. doi:10.1104/pp.103.020347. 12857824 PMC167082

[33] Dekkers BJ, Schuurmans JA, Smeekens S. Interaction between sugar and abscisic acid signalling during early seedling development in Arabidopsis. Plant Mol Biol. 2008;67(1–2):151–167. doi:10.1007/s11103-008-9308-6. 18278579 PMC2295253

[34] Favery B, Ryan E, Foreman J, Linstead P, Boudonck K, Steer M, Dolan L, Dolan L. KOJAK encodes a cellulose synthase-like protein required for root hair cell morphogenesis in Arabidopsis. Genes Dev. 2001;15(1):79–89. doi:10.1101/gad.188801. 11156607 PMC312599

[35] Howles PA, Birch RJ, Collings DA, Gebbie LK, Hurley UA, Hocart CH, Arioli T, Williamson RE. A mutation in an Arabidopsis ribose 5‐phosphate isomerase reduces cellulose synthesis and is rescued by exogenous uridine. Plant J. 2006;48(4):606–618. doi:10.1111/j.1365-313X.2006.02902.x. 17059404

[36] Singh SK, Fischer U, Singh M, Grebe M, Marchant A. Insight into the early steps of root hair formation revealed by the *procuste1* cellulose synthase mutant of Arabidopsis thaliana. BMC Plant Biol. 2008;8(1):57. doi:10.1186/1471-2229-8-57. 18485206 PMC2396171

[37] Tsang DL, Edmond C, Harrington JL, Nühse TS. Cell wall integrity controls root elongation via a general 1-aminocyclopropane-1-carboxylic acid-dependent, ethylene-independent pathway. Plant Physiol. 2011;156(2):596–604. doi:10.1104/pp.111.175372. 21508182 PMC3177261

[38] Sieberer BJ, Ketelaar T, Esseling JJ, Emons AM. Microtubules guide root hair tip growth. New Phytol. 2005;167(3):711–719. doi:10.1111/j.1469-8137.2005.01506.x. 16101908

[39] Šamaj J, Müller J, Beck M, Böhm N, Menzel D. Vesicular trafficking, cytoskeleton and signalling in root hairs and pollen tubes. Trends Plant Sci. 2006;11(12):594–600. doi:10.1016/j.tplants.2006.10.002. 17092761

[40] Molendijk AJ, Bischoff F, Rajendrakumar CS, Friml J, Braun M, Gilroy S, Palme K. *Arabidopsis thaliana* Rop GTPases are localized to tips of root hairs and control polar growth. Embo J. 2001;20(11):2779–2788. doi:10.1093/emboj/20.11.2779. 11387211 PMC125484

[41] Jones MA, Shen JJ, Fu Y, Li H, Yang Z, Grierson CS. The Arabidopsis Rop2 GTPase is a positive regulator of both root hair initiation and tip growth. Plant Cell. 2002;14(4):763–776. doi:10.1105/tpc.010359. 11971133 PMC150680

[42] Bloch D, Lavy M, Efrat Y, Efroni I, Bracha-Drori K, Abu-Abied M, Yalovsky S, Yalovsky S. Ectopic expression of an activated RAC in Arabidopsis disrupts membrane cycling. Mol Biol Cell. 2005;16(4):1913–1927. doi:10.1091/mbc.e04-07-0562. 15703216 PMC1073671

[43] Yang G, Gao P, Zhang H, Huang S, Zheng ZL, Grebe M. A mutation in MRH2 kinesin enhances the root hair tip growth defect caused by constitutively activated ROP2 small GTPase in Arabidopsis. PLoS One. 2007;2(10):e1074. doi:10.1371/journal.pone.0001074. 17957256 PMC2031828

[44] Lombardo MC, Graziano M, Polacco JC, Lamattina L. Nitric oxide functions as a positive regulator of root hair development. Plant Signal Behav. 2006;1(1):28–33. doi:10.4161/psb.1.1.2398. 19521473 PMC2633697

[45] Lombardo MC, Lamattina L. Nitric oxide is essential for vesicle formation and trafficking in Arabidopsis root hair growth. J Ext Bot. 2012;63(13):4875–4885. doi:10.1093/jxb/ers166. 22791827

[46] Pasrija R, Thakur JK. Analysis of differential expression of Mediator subunit genes in Arabidopsis. Plant Signal Behav. 2012;7(12):1676–1686. doi:10.4161/psb.22438. 23072992 PMC3578909

[47] Esparza-Reynoso S, Ruiz-Estrella LF, Pelagio-Flores R, Macias-Rodríguez LI, Martínez-Trujillo M, López-Coria M, Sánchez-Nieto S, Herrera-Estrella A, López-Bucio J. Trichoderma atroviride -emitted volatiles improve growth of Arabidopsis seedlings through modulation of sucrose transport and metabolism. Plant Cell Environ. 2021;44(6):1961–1976. doi:10.1111/pce.14014. 33529396

[48] Stadler R, Wright KM, Lauterbach C, Amon G, Gahrtz M, Feuerstein A, Oparka KJ, Sauer N. Expression of GFP-fusions in Arabidopsis companion cells reveals non-specific protein trafficking into sieve elements and identifies a novel post-phloem domain in roots. Plant J. 2005;41(2):319–331. doi:10.1111/j.1365-313X.2004.02298.x. 15634207

